# Deletion of Irs2 causes reduced kidney size in mice: role for inhibition of GSK3β?

**DOI:** 10.1186/1471-213X-10-73

**Published:** 2010-07-06

**Authors:** Rosemarie M Carew, Marianna Sadagurski, Roel Goldschmeding, Finian Martin, Morris F White, Derek P Brazil

**Affiliations:** 1 UCD Diabetes Research Centre, UCD Conway Institute, School of Medicine and Medical Science, University College Dublin, Belfield Dublin 4, Ireland; 2 UCD Conway Institute, School of Biomolecular and Biomedical Science, University College Dublin, Belfield Dublin 4, Ireland; 3 Centre for Vision and Vascular Science, School of Medicine, Dentistry and Biomedical Science, Queen's University Belfast, Belfast BT12 6BA, Northern Ireland, UK; 4 Howard Hughes Medical Institute, Division of Endocrinology, Children's Hospital Boston, Harvard Medical School, Boston, MA 02115, USA; 5 Department of Pathology, University Medical Center Utrecht, Heidelberglaan 100, 3584 CX Utrecht, The Netherlands

## Abstract

**Background:**

Male *Irs2*^-/- ^mice develop fatal type 2 diabetes at 13-14 weeks. Defects in neuronal proliferation, pituitary development and photoreceptor cell survival manifest in *Irs2*^-/- ^mice. We identify retarded renal growth in male and female *Irs2*^-/- ^mice, independent of diabetes.

**Results:**

Kidney size and kidney:body weight ratio were reduced by approximately 20% in *Irs2*^-/- ^mice at postnatal day 5 and was maintained in maturity. Reduced glomerular number but similar glomerular density was detected in *Irs2*^-/- ^kidney compared to wild-type, suggesting intact global kidney structure. Analysis of insulin signalling revealed renal-specific upregulation of PKBβ/Akt2, hyperphosphorylation of GSK3β and concomitant accumulation of β-catenin in *Irs2*^-/- ^kidney. Despite this, no significant upregulation of β-catenin targets was detected. Kidney-specific increases in Yes-associated protein (YAP), a key driver of organ size were also detected in the absence of *Irs2*. YAP phosphorylation on its inhibitory site Ser127 was also increased, with no change in the levels of YAP-regulated genes, suggesting that overall YAP activity was not increased in *Irs2*^-/- ^kidney.

**Conclusions:**

In summary, deletion of *Irs2 *causes reduced kidney size early in mouse development. Compensatory mechanisms such as increased β-catenin and YAP levels failed to overcome this developmental defect. These data point to *Irs2 *as an important novel mediator of kidney size.

## Background

The insulin receptor substrate (IRS) family of proteins plays a key role in insulin signalling [1]. Most, if not all, insulin signals are modulated through tyrosine phosphorylation of IRS proteins [2]. IRS proteins are intermediate cellular scaffold proteins, which act as an interface between tyrosine kinase receptors and effector proteins such as PI3K and MAPK. The crucial role of IRS proteins in insulin action has been demonstrated using knockout mouse models [3]. The distinct phenotypes of *Irs1*^-/- ^and *Irs2*^-/- ^mice present a fascinating physiological contrast, despite the significant structural similarities of these proteins [4]. *Irs1*^-/- ^mice are approximately 50% smaller in size compared to littermate controls, highlighting the importance of IRS1 in somatic growth promotion [5-7]. *Irs1*^-/- ^mice are insulin resistant but also maintain normal fasting glucose and glucose tolerance due to compensatory β-cell hyperplasia [5,6]. In contrast, mice lacking *Irs2 *develop type 2 diabetes, with *Irs2*^-/- ^male mice displaying both insulin resistance and β-cell failure at an early age [8,9]. In light of their pivotal role in insulin signalling, IRS proteins have been examined as candidate genes for type 2 diabetes and other human metabolic disorders [1].

Animal and organ size is determined by hormones and growth factors that control processes such as cell number, cell size and cell death during fetal development and postnatal growth [10-12]. The important role of the insulin/IGF-1 signalling pathway in growth and development is well established. Ablation of insulin and IGF-1 receptors results in early postnatal death [13-15]. IRS2 does not regulate somatic growth to the same extent as IRS1, and *Irs2*^-/- ^mice were only 10% smaller in size compared to wild-type controls [7]. However, *Irs2 *gene deletion precipitates a number of pathophysiological effects in organs that are typically affected by diabetic complications. In the retina, *Irs2 *deletion in mice caused increased photoreceptor cell apoptosis, with decreased maturation and survival of photoreceptors [16]. *Irs2*^-/- ^mice also display reduced neuronal proliferation, leading to reduced brain size [17]. In the kidney, insulin is known to modify several functions of renal tubules [18-23], with IRS2 playing a major role in insulin stimulation of renal proximal tubular transport and PKB/Akt phosphorylation *in vitro *[24]. Since insulin-like, and other growth factor signalling is crucial in organ development [25], we examined the effect of *Irs2 *gene deletion on kidney function and development in mice. Our data highlight a novel role for IRS2 signalling in kidney size development and insulin signalling in renal cells.

## Results

### IRS2 is expressed in mouse kidney

**Figure 1:**
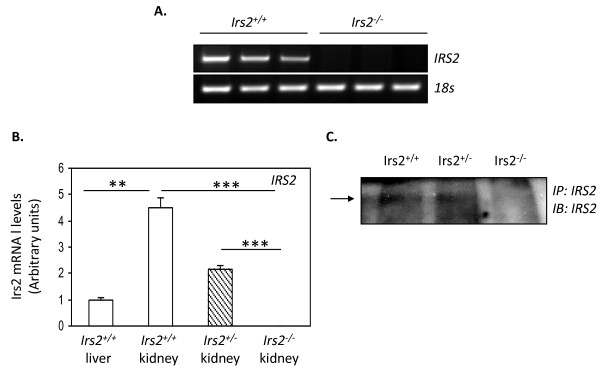
**IRS2 is detected in mammalian kidney**. Semi-quantitative (A) and quantitative TaqMan (B) PCR was performed using RNA from renal poles of 5-6 wk *Irs2*^+/+ ^*(empty bars), Irs2*^+/- ^*(striped bars*) and *Irs2*^-/- ^mice *(filled bars) and Irs2 *specific oligonucleotides as described in Material and Methods. ΔΔCt values were calculated by subtracting the Ct values for IRS2 by those for the 18S control performed simultaneously, and fold change was then calculated by setting the wild-type liver group value to 1. Data are expressed as mean +/- SEM, n = 5 for each group. Statistical significance was determined using one way ANOVA with post hoc Tukey-Kramer multiple comparisons test, *** p < 0.001. (C) Immunoprecipitation was carried out on 1 mg protein lysates from *Irs2*^+/+^, *Irs2*^+/- ^and *Irs2*^-/- ^kidney. Protein lysates were separated on 7.5% SDS-PAGE and probed by Western blotting with anti-IRS2 antibody.

Levels of IRS2 mRNA were high in mouse kidney compared to mouse liver (Fig. 1A, B). IRS2 protein was detected in wild-type and *Irs2*^+/- ^kidney, but not in *Irs2*^-/- ^kidney extracts (Fig. 1C). A modest 2-fold increase in IRS1 mRNA levels was detected in *Irs2*^-/- ^kidney, suggesting a mild compensatory increase in IRS1 in the absence of IRS2 (Additional File 1 Fig. S1).

### Deletion of Irs2 causes decreased kidney size in mice

**Figure 2:**
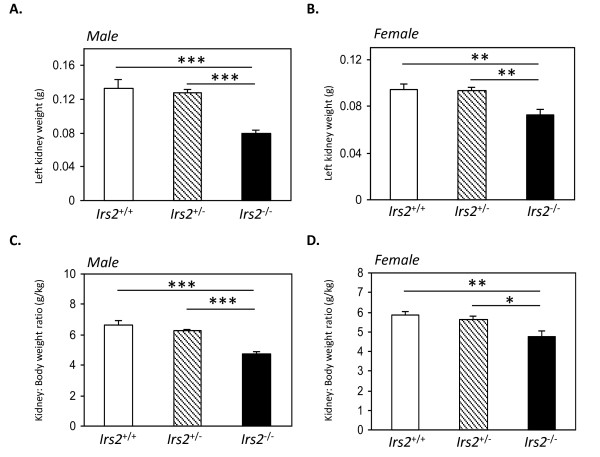
***Irs2 *gene deletion significantly decreases kidney-to-body weight ratio**. Left kidney weights of 5-6 wk old (A) male and (B) female *Irs2*^+/+ ^(open bars), *Irs2*^+/- ^(hatched bars) and *Irs2*^-/- ^(filled bars) mice were measured at the time of sacrifice. Kidney-to-body weight ratios were calculated for (C) male and (D) female groups and expressed as g/kg. Data were plotted as mean +/- SEM (n = 7-8 for each group).

**Figure 3:**
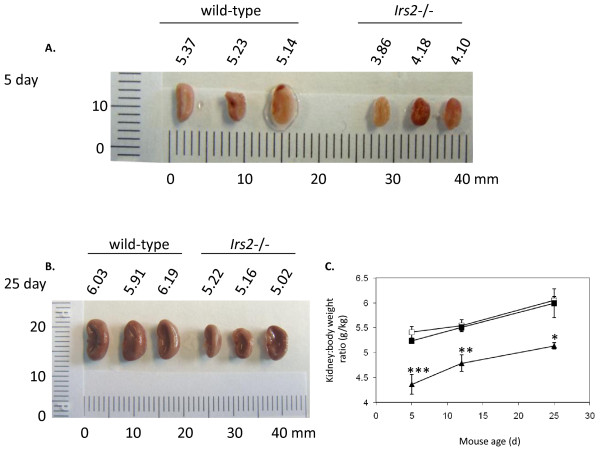
**Kidney size is reduced at early postnatal timepoints in *Irs2*-/- mice**. Kidneys (n = 3) from (A) 5 d and (B) 25 d wild-type and Irs2-/- male and female mice were removed upon sacrifice and photographed as described. Size in mm is shown for scale. (C) Left kidney-to-body weight ratios were calculated for *Irs2*^+/+ ^(*open square*s), *Irs2*^+/- ^(*filled squares*) and *Irs2*^-/- ^(*filled triangles*) mice at 5 d, 12 d and 25 d male and female mice combined and expressed as g/kg. Data were plotted as mean +/- SEM, n = 3-5 for each group. Statistical significance was determined using one-way ANOVA and Tukey-Kramer multiple comparison test, * p < 0.05, ** p < 0.01, *** p < 0.001.

Both kidney weight and kidney-to-body weight ratio were reduced in *Irs2*^-/- ^mice at 5-6 wk of age, in both male and female mice (Fig. 2 and Additional File 1 Table S1). Kidney size was clearly smaller in *Irs2*-/- mice compared to wild-type at various time-points (Fig. 3A, B and Additional File 1 Fig. S2). Newborn *Irs2*^-/- ^mice displayed a reduction in kidney-to-body weight ratio at 5 d of age (*wild-type 5.411 +/- 0.115*, Irs2-/- *4.364 +/- 0.2, p < 0.001*) which was maintained up to 25 d and beyond (Fig. 3C). The slopes of the growth curves indicated that *Irs2*^-/- ^mice had a higher rate of growth between 5 d and 12 d compared to wild-type or *Irs2*^+/- ^(*Irs2*^+/+ ^*slope 0.0189, Irs2*^-/- ^*slope 0.0605*). However, the growth rate was similar between 12 d and 25 d in all three genotypes, suggesting that this higher rate of growth was not maintained in *Irs2*^-/- ^mice. Supporting this observation, kidney size in older female mice (43-44 wk) remained smaller in *Irs2*^-/- ^mice compared to wild-type (data not shown), suggesting that the initial defect in kidney growth is not overcome during the lifetime of the *Irs2*^-/- ^mouse. No significant histological changes were seen in *Irs2*^-/- ^kidneys compared to wild-type at any age group, using a range of specific stains for markers of kidney damage (Additional File 1 Fig. S3). Quantitation of glomeruli in H&E stained paraffin sections revealed that *Irs2*^-/- ^kidneys contained less glomeruli per kidney section than wild-type (Irs2-/- *200+/- 29 glomeruli per section, wild-type 217+/- 25 glomeruli per section*). However, when normalised for sectional area, a similar density of glomeruli (and by inference, nephrons) was present in *Irs2*-/- kidneys compared to wild-type mice (Additional File 1 Fig. S4). Together, these data suggest that *Irs2 *deletion leads to a global reduction in mouse kidney size during development.

### *Irs2 *deletion causes increased PKB/Akt signalling specifically in kidney

**Figure 4:**
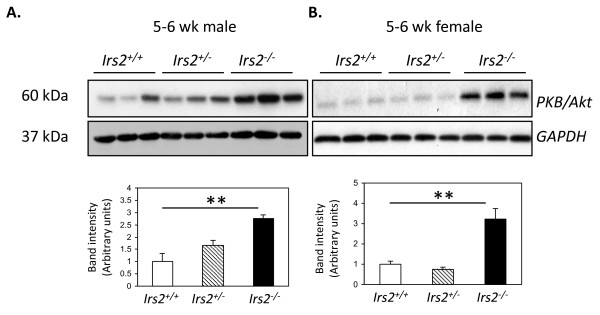
**Increased PKB/Akt expression in *Irs2*^-/- ^mouse kidney**. Twenty μg of kidney protein lysates from 5-6 wk *Irs2*^+/+^, *Irs2*^+/- ^and *Irs2*^-/- ^5-6 wk male (A) and female (B) groups were separated on 10% SDS-PAGE and probed by Western blotting with anti-PKB/Akt. GAPDH was used as a loading control. Band intensities were calculated using Scion Image software. The intensity ratios of PKB/GAPDH are shown. ** p < 0.01.

**Figure 5:**
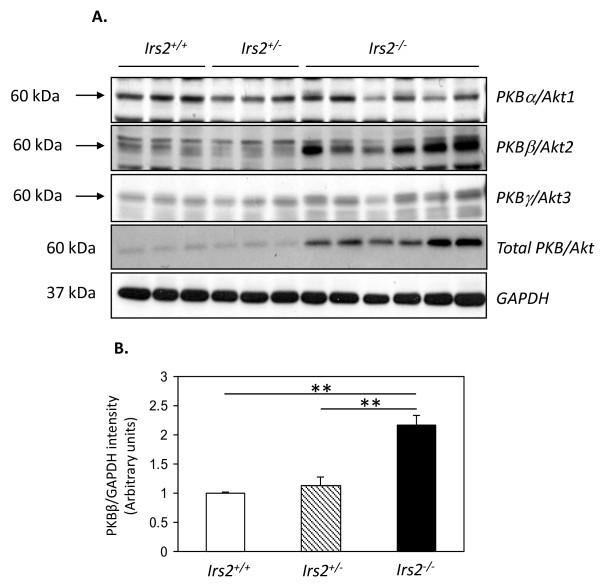
**Specific increase in PKBβ/Akt2 is detected in Irs2-/- kidney**. (A) Twenty μg of protein lysates from *Irs2*^+/+ ^(n = 3), *Irs2*^+/- ^(n = 3) and *Irs2*^-/- ^(n = 6) mice were separated on 10% SDS-PAGE and probed by Western blotting with anti-PKBα/Akt1, PKBβ/Akt2, PKBγ/Akt3 and anti-PKB/Akt antibodies. GAPDH was used as a loading control. (B) Band intensities were quantified using Scion Image software. The intensity ratio of anti-PKBβ/GAPDH is shown. ** p < 0.01.

PKB/Akt activation is a key regulator of glomerular podocyte and tubular epithelial cell survival [26-29]. The status of PKB/Akt expression was therefore assessed in kidneys of wild-type, *Irs2*+/- and *Irs2*-/- kidneys. Surprisingly, levels of total PKB/Akt were elevated in both male and female *Irs2*-/- kidneys at 5-6 wk of age compared to wild-type and *Irs2*^+/- ^(Fig. 4). This 2.5-3-fold increase in PKB/Akt expression was also maintained in older mice (data not shown). Using isoform specific antibodies, marked upregulation of PKBβ/Akt2 was identified in *Irs2*^-/- ^mouse kidney compared to controls (Fig. 5). This increase was not observed in *Irs2*^-/- ^liver or brain (data not shown), suggesting that upregulation of PKBβ/Akt2 may be specific to *Irs2*^-/- ^mouse kidney. No change in the level of mRNA for all three PKB/Akt isoforms was detected in *Irs2*^-/- ^kidney compared to wild-type or *Irs2*^+/-^, suggesting that either increased PKBβ/Akt2 translation or post-transcriptional stability may account for the observed protein increase in kidney in the absence of *Irs2*.

**Figure 6:**
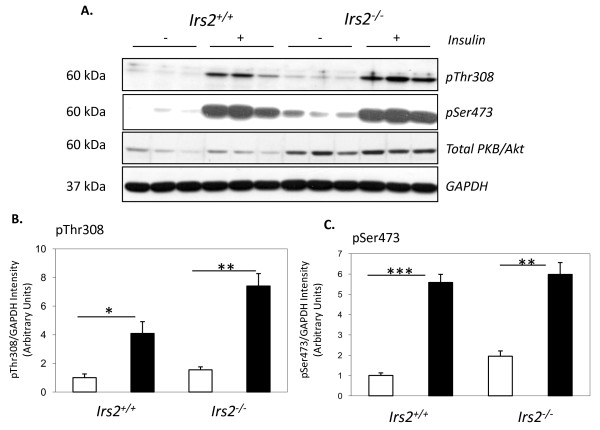
**Increased insulin-stimulated PKB/Akt activation in Irs2-/- kidney**. (A) Twenty μg of kidney lysates from vehicle ("-") and insulin("+") -injected 5-6 wk *Irs2*+/+ and *Irs2*-/- mice were separated on 10% SDS-PAGE and probed by Western blotting with anti-phospho-PKB/Akt Thr308, anti-phospho-PKB/Akt Ser473 and anti-PKB/Akt. GAPDH was used as a loading control. (B, C) Band intensities were quantified using Scion Image software. The intensity ratio of pThr308/GAPDH and pSer473/GAPDH are shown. * p < 0.05, ** p < 0.01, ***p < 0.001.

To explore whether defects in growth factor signalling contribute to decreased kidney size in *Irs2*-/- mice, the activation of PKB/Akt in kidney in response to insulin was assessed. Insulin stimulated PKB/Akt phosphorylation in both wild-type and *Irs2*^-/- ^kidney (Fig. 6A-C). Levels of basal PKB/Akt Thr308 and Ser473 phosphorylation also appeared to be higher in *Irs2*^-/- ^kidney compared to wild-type (Fig. 6). Insulin injection triggered a higher degree of PKB/Akt phosphorylation on Thr308 in *Irs2*^-/- ^kidney compared to wild-type, whereas fold-change of Ser473 phosphorylation was similar between the two genotypes (Fig. 6). These data suggest that PKB/Akt activation may be increased in the absence of IRS2 in kidney.

**Figure 7:**
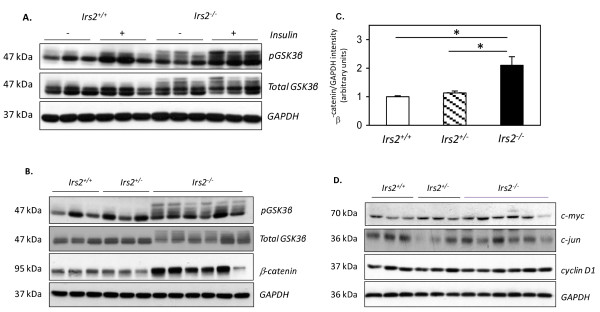
**Altered GSK3β/β-catenin signalling is evident in *Irs2*^-/- ^mouse kidney**. (A). Twenty μg of kidney protein lysates from Irs2+/+ and Irs2-/- mice injected with insulin were probed by Western blotting with the indicated antibodies. (B) Twenty μg of kidney protein lysates from female *Irs2*^+/+ ^(n = 3), *Irs2*^+/- ^(n = 3) and *Irs2*^-/- ^(n = 6) mice were probed with the indicated antibodies. GAPDH was used as loading control. (C) Band intensities were quantified using Scion Image software. The intensity ratio of β-catenin/GAPDH is shown. p *< 0.05. (D) Twenty μg of kidney protein lysates from *Irs2*^+/+ ^(n = 3), *Irs2*^+/- ^(n = 3) and *Irs2*^-/- ^(n = 6) mice were probed with anti-c-myc and anti-cyclin D1 antibodies.

The effect of increased PKBβ/Akt2 expression on the phosphorylation of GSK3β, a major downstream target of PKB/Akt was then assessed. Phosphorylation of GSK3β on the inhibitory Ser9 site increased in both wild-type and *Irs2*^-/- ^kidney in response to insulin (Fig. 7A). However, a number of additional bands were detected with both phospho- and total GSK3β antibodies in *Irs2*^-/- ^kidney extracts compared to wild-type, in both vehicle and insulin-stimulated mice (Fig. 7A). These data suggest that increased PKB/Akt activation triggers hyperphosphorylation and inactivation of GSK3β in kidney. Consistent with this hypothesis, levels of β-catenin, a protein targeted by GSK3β for degradation, were markedly elevated in *Irs2*^-/- ^kidney compared to controls (Fig. 7B, C). However, despite increased accumulation of β-catenin, no major changes in the expression of β-catenin targets c-myc, c-jun and cyclin D1 were evident in *Irs2*^-/- ^kidneys (Fig. 7D).

### Deletion of *Irs2 *causes kidney-specific increases in YAP expression and phosphorylation

**Figure 8:**
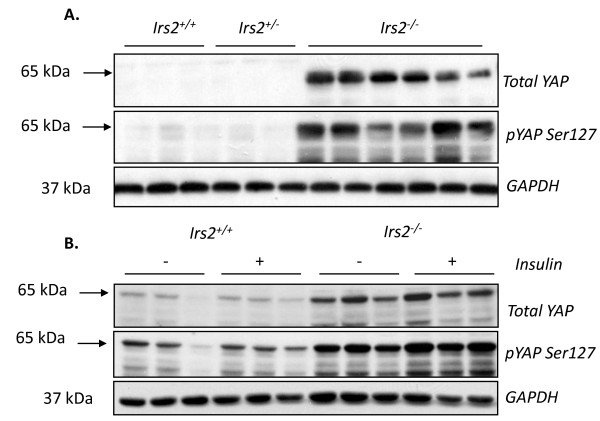
**Phosphorylated and total YAP levels are markedly elevated in *Irs2*^-/- ^mouse kidney**. (A) Twenty μg of kidney protein lysates from *Irs2*^+/+ ^(n = 3), *Irs2*^+/- ^(n = 3) and *Irs2*^-/- ^(n = 6) mice probed by Western blotting as indicated. (B) Six 5-6 wk female *Irs2*^+/+ ^and *Irs2*^-/- ^mice were injected with insulin as indicated in Materials and Methods. Twenty μg of kidney protein lysates were probed as in (A). GAPDH was used as a loading control.

The Hippo-YAP pathway has shown to be crucial in cellular processes that increase mammalian organ size [30-32]. YAP is a transcriptional co-activator that is phosphorylated by kinase cascades such as Hippo/Mst1, leading to cytoplasmic localisation and inhibition of transcription [32,33]. Levels of total YAP were almost undetectable in kidney extracts of adult wild-type mice, but were significantly elevated in *Irs2*^-/- ^kidney compared to controls (Fig. 8A). Importantly, levels of YAP phosphorylation on the inhibitory site Ser127 were also increased in the absence of Irs2 (Fig. 8A). Previous data have suggested that PKB/Akt may phosphorylate YAP, inhibiting its function [33]. In our study, insulin treatment had little effect on YAP phosphorylation in either wild-type and *Irs2*^-/- ^kidney (Fig. 8B). No changes in YAP levels or phosphorylation were detected in other mouse tissues (data not shown). These data suggest that deletion of *Irs2 *causes increased expression of the transcriptional co-activator YAP in kidney, which is immediately inactivated by phosphorylation, preventing potential YAP-induced increases in kidney size.

## Discussion

IRS2 is a fundamental element in normal insulin and IGF-1 signalling, regulating glucose metabolism, brain growth, ovarian development and retinal photoreceptor apoptosis [7,9,16,17,34]. Defects in insulin signalling may contribute to diabetic kidney disease [25]. Our data identify a novel role for IRS2 in renal development and in the regulation of PKB/Akt/GSK3β and Hippo/YAP signalling in the kidney.

Defects in organ growth and development have been reported due to *Irs2 *gene deletion [16,17], with IRS1 playing a more dominant role in somatic growth compared to IRS2 [5-7]. Expression of IRS2 can be detected in renal cortex at E14.5, with minimal IRS1 and IRS3 detecetd at the same developmental time-point http://www.eurexpress.org. Smaller kidneys were evident in *Irs2*^-/- ^mice as early as postnatal day 5 (Fig. 2, 3), and kidney-to-body weight ratio increased more rapidly between postnatal day 5 and 12 in *Irs2*^-/- ^mice compared to wild-type, suggestive of catch-up growth in these animals (Fig. 3C). However, this increased rate of growth was not sustained, and the kidney size defect did not recover as *Irs2*^-/- ^mice aged. Interestingly, it has been reported that kidney and brain from *Irs1*^-/- ^mice exhibited proportionately smaller weight reductions than the decrease in body weight [35], as opposed to larger kidney/organ-to-body weight ratio reduction observed in *Irs2*^-/- ^mice. These data emphasise the predominant role of IRS2 rather that IRS1 in determining the growth of organs such as kidney and brain.

Despite decreased kidney size in *Irs2*^-/- ^mice, no major morphological changes or reduction in nephron density were detected, apart from slight compensatory glomerular hypertrophy. These results add to previous knockout studies which demonstrated that insulin/IGF-1 signalling pathway is not essential for renal morphogenesis, but may be important for overall organ growth [13-15].

The kidney-specific upregulation of PKBβ/Akt2 in *Irs2*^-/- ^mice is interesting based on the overall similarity of PKB/Akt isoforms, and previous data demonstrating a degree of redundancy within the PKB/Akt family [36]. Mice lacking Rictor, a key element of the TORC2 complex shown to phosphorylate PKB/Akt on Ser473 [37], develop increased organ size, including kidney [38]. The higher basal level of Ser473 phosphorylation seen in *Irs2*-/- kidney suggests that higher TORC2 activity may contribute to reduced kidney size in the absence of IRS2. Experiments carried out by Zheng and colleagues identified that deletion of *Irs2 *attenuated insulin stimulated bicarbonate absorption in isolated renal proximal tubules [24]. Of note, these authors detected a decrease in PKB/Akt phosphorylation on Ser473 with no apparent change in PKB/Akt expression in kidney cortex of *Irs2*^-/- ^mice (Fig. 6A, C[24]). The divergence between these observations and our data presented here may be explained by the fact that our study examined whole kidney lysates rather than isolated, cultured renal cortex slices. In contrast to Zheng et al who examined Ser473 phosphorylation alone, the increase in PKB/Akt phosphorylation was mainly detected on Thr308 in our experiments, and was supported by increased GSK3β phosphorylation on Ser9 (Fig. 6, 7).

The specific increase in PKBβ/Akt2 protein (but not mRNA) suggests increased translation of PKBβ/Akt2 mRNA, or that posttranslational processes leading to PKBβ/Akt2 degradation are absent in the *Irs2*-/- kidney. This novel finding also suggests that this process is specific for the PKBβ/Akt2 isoform. This increase in expression, together with increased PKB/Akt phosphorlyation in the basal state may represent a compensatory mechanism resulting from the loss of IRS2, to enhance growth factor signalling in an effort to overcome defects in kidney growth during development. Importantly, these data imply that, contrary to the predicted cellular effects, loss of IRS2 somehow enhances, rather than inhibits PKB/Akt activation in the kidney. The precise molecular consequences of *Irs2 *deletion in kidney cells remain to be determined.

GSK3β is directly inactivated downstream of PKB/Akt via phosphorylation on Ser9 [39,40]. Hyperphosphorylation of GSK3β was detected in *Irs2*^-/- ^kidney but not in liver or brain (Fig. 7). The "laddering" of GSK3β bands reactive with the phospho-Ser9 antibody, together with a decrease in the intensity of the GSK3β band at 47 kDa (c.f. Fig. 7B) suggest that deletion of *Irs2 *causes PKB/Akt phosphorylation-dependent decreases in GSK3β activity. The accumulation of β-catenin (a GSK3β target) in the kidney of *Irs2*^-/- ^mice (Fig 7B, C) suggests that increased phosphorylation of GSK3β leads to reduced GSK3β kinase activity in the kidney, facilitating β-catenin accumulation. This increase in β-catenin was not detected in other tissues; again suggesting that defects in PKB/Akt/GSK3β/β-catenin pathway is kidney specific in *Irs2*^-/- ^mice. Active β-catenin binds to the TCF/Lef family of transcription factors [41] and serves as a co-activator to stimulate the transcription of genes such as cyclin D1 and c-myc [42,43], which drive cell proliferation. However, protein levels of these β-catenin targets were not significantly altered in *Irs2*^-/- ^kidney versus controls (Fig. 7D). Thus, increased cellular β-catenin levels do not appear to be sufficient to increase protein targets in *Irs2*^-/- ^kidney. It remains to be determined if changes in β-catenin levels alter its transcriptional activity during a period of defective embryonic kidney growth during development. Significantly, insulin-stimulated PKB/Akt activation was not decreased in *Irs2*-/- kidney at 5-6 wk (Fig. 6), and no changes in basal PKB/Akt/GSK3β signalling were detected in 5-6 wk *Irs2*-/- liver, where profound insulin resistance was previously reported (9). We therefore hypothesise that signalling changes reported here occur as a direct result of *Irs2 *deletion in kidney, and not as a result of insulin resistance. Analysis of PKB/Akt signalling at earlier time-points will help clarify these issues.

Several studies have clearly established that Hippo/Mst1 pathway is critically involved in regulating organ growth [30-32]. In mammals, the Mst1/LATS/Mob1/YAP pathway plays a key role in restricting organ size by controlling both cell proliferation and apoptosis [44-46]. The final component of this pathway, YAP [47], is a potent growth promoter, and overexpression of YAP in the liver leads to organ enlargement [30,31]. YAP is inhibited by the Hippo pathway via phosphorylation on Ser127, leading to its cytoplasmic translocation and retention [32]. YAP levels were markedly elevated in *Irs2*^-/- ^kidney compared to wild-type controls (Fig. 8A, B) Even though levels of YAP were higher in *Irs2*^-/- ^kidney, an increase in YAP phosphorylation on Ser127 was also evident, suggesting that immediate inactivation and nuclear export of YAP may occur in *Irs2*-/- kidney cells, preventing increased kidney growth. Preliminary data from our laboratory suggests that levels of active Mst1, the upstream kinase responsible for YAP phosphorylation on Ser127, are also elevated in *Irs2*^-/- ^kidney. Consistent with these findings, protein levels of YAP downstream targets, E-cadherin and survivin did not change significantly in *Irs2*^-/- ^kidney (data not shown). Therefore, similar to increased β-catenin, the accumulation of YAP in adult kidney may represent a compensatory mechanism for reduced kidney size in *Irs2*^-/- ^mice. Ultimately, this compensatory response is ineffective due to rapid Ser127 phosphorylation and therefore inactivation of YAP. Thus, we hypothesise that reduced kidney size in *Irs2*-/- may trigger changes in GSK3β and YAP signalling that ultimately fail to overcome the growth defect in kidney.

## Conclusions

In summary, we have established that mice lacking *Irs2 *display defects in kidney growth and PKB/Akt/GSK3β signalling. It is clear that the integration of many signalling pathways is required to regulate normal organ development. *Irs2 *gene deletion results in decreased kidney size and our observations suggest that specific signalling changes in the PKB/Akt and YAP pathway occur in *Irs2*-/- kidney. Future experiments will strive to identify the organ-specific growth control pathways that are relevant to kidney development in the *Irs2*^-/- ^mouse.

## Methods

### Experimental Animals

All mouse experiments were carried out in accordance with the European Communities Council Directive (86/609/EEC). All mouse handling was performed by licensed technicians in the University College Dublin Biomedical Facility and was performed with the appropriate governmental and institutional ethical and legal approval and licenses. Experimental animals were generated by crossing *Irs2*^+/- ^mice on a mixed genetic background (C57Bl/6 × 129sv). Genotyping was performed using DNA extracted from ear punches as described [9]. Anaesthesia and perioperative analgesia were used to maintain animal well-being throughout the experiments.

### *In vivo *manipulations

All biochemical analysis was carried out according to Animal Models of Diabetic Complications Consortium, http://www.amdcc.org. Body weight was assessed biweekly until time of harvest. For insulin injections, groups of *Irs2*^+/+ ^and *Irs2*^-/- ^mice were starved overnight and injected intraperitoneally (i.p.) with 5 U human insulin (Humulin S, Eli Lilly and Co.) or saline vehicle for 30 min, after which mice were sacrificed by cervical dislocation and tissues harvested.

### Tissue/sample preparation

The left kidney was ligated, removed and snap-frozen for protein and RNA extraction. Images were captured by thawing whole kidneys from both wild-type and *Irs2*-/- mice (stored in RNAlater) and photographing on a glass slide. Perfusion-fixation of the right kidney was performed using non-fixative sterile normal saline (pH 7.4) for 5 min, followed by 4% (w/v) paraformaldehyde (pH 7.4) for 5 min. The perfused right kidney was then removed and submersed in 4% paraformaldehyde for 24 h at RT. Kidneys were then paraffin embedded, cut at 3 μm thickness and stained with haematoxylin eosin staining, Masson's Trichrome, periodic acid-Schiff and Picrosirius Red. Slides were scanned at 40 × magnification using a ScanScope XT slide scanner (Aperio Technologies). Stained sections were scored independently (single blinded) by pathologists at the Department of Pathology, University Medical Center Utrecht, Netherlands. For calculation of glomerular number, each kidney was divided in 4 parts of equal thickness, of which the central two sections were embedded on successive non-adjacent cut-surfaces. Regions of the renal cortex were selected and the number of glomeruli counted in each square. This was repeated for 3 sections per kidney in 3 mice from 5-6 wk male *Irs2*^+/+ ^and *Irs2*^-/- ^groups. Glomeruli number was then normalised to sectional area in each group.

### IRS2/IRS1 RNA measurements

Total RNA was extracted from cells and mouse tissues using Trizol (R) Reagent from Invitrogen. RNA from tissues was further purified using RNeasy columns (Qiagen). Reverse transcription reactions were performed using 1 μg of RNA and Superscript™ II (Invitrogen). One μl of synthesised cDNA was then used for semi-qauntitative and TaqMan PCR to amplifiy either IRS1 or IRS2. Oligonucleotides for semiquantitative PCR were as described (Withers et al 1998) and for TaqMan were purchased from Applied Biosystems (IRS2, Mm03038438_m1; IRS1 Mm00439720_s1). 18S was run as a normalisation control and the ΔΔCt method was used to calculate fold-change in RNA levels for each gene.

### Protein extraction, immmunoblotting and immunoprecipitation

Both kidney tissue and cultured cells were lysed in RIPA buffer exactly as previously described [29]. Antibodies at the following dilutions were used: IRS-2 (1:500 generated in-house, Prof. Morris White), IRS-2 (Millipore, used for immunoprecipitation), polyclonal pAkt Ser473 (1:1000, Cell Signaling), pAkt Thr 308 (1:1000, Cell Signaling), pGSK-3β Ser9 (1:1000, Cell Signaling), total GSK-3β (1:1000, Cell Signaling), β-catenin (1:1000, BD Biosciences), total YAP (1:1000, Cell Signaling), pYAP Ser127 (1:1000, Cell Signaling), isoform specific PKB/Akt antibodies PKBα (1:500), PKBβ (1:500), PKBγ (1:5000) were a generous gift from Dr. Brian Hemmings, Friedrich Miescher Institute, Basel, Switzerland, total PKB/Akt (1:1000, Cell Signaling), GAPDH (1:5000, Cell Signaling), β-actin (1:25,000, Sigma). Reactive bands were revealed using HRP-coupled secondary antibodies and enhanced chemiluminescence reagents (Santa Cruz Biotechnology) and X-ray film.

Immunoprecipitation was carried out to detect IRS2 expression in the kidney. Kidneys were lysed as described above. Supernatants containing 1 mg of protein lysate were pre-cleared with 20 μl of a 50% slurry of protein-A/G-Sepharose (Pierce) for 60 min at 4°C and incubated overnight with 5 μl (5 μg) anti-IRS2 antibody (Millipore). Immune complexes were collected with 20 μl of a 50% slurry of protein-A/G-Sepharose and resolved on a 7.5% SDS-PAGE as described above.

### Statistical analysis

All data were expressed as mean +/- SEM. Statistical analysis was carried out using InStat software. Student's unpaired t-test or analysis of variance (ANOVA) with post hoc Tukey-Kramer Multiple Comparison test was used, with a difference of p < 0.05 considered significant.

## Authors' contributions

RC carried out the experiments presented, MS provided insulin-stimulated kidneys for pilot analysis, RG performed histological scoring of kidney sections, FM provided expert advice and MFW provided *Irs2*+/- mice, antibody reagents and expert advice. DPB performed some experiments and wrote the final manuscript. All authors read and approved the final manuscript.

## Supplementary Material

Additional file 1 **Elevated IRS1 gene expression in the kidneys of male 13-14 wk *Irs2*^-/- ^diabetic mice**. Compensatory changes in kidney IRS1 in the absence of IRS2. Click here for file

Additional file 2 **Reduced kidney size is evident in *Irs2*-/- kidneys compared to wild-type at 12 days of age**. Kidney size is reduced in *Irs2*-/- mice at 12 d of age. Click here for file

Additional file 3 ***Irs2 *deletion does not significantly alter gross kidney structure**. Histology of Irs2-/- kidneys showing no significant changes in morphology. Click here for file

Additional file 4 ***Irs2*^-/- ^mice have reduced glomerular number but normal nephron density**. No major changes in glomerular density in Irs2-/- kidneys Click here for file

Additional file 5 **Kidney: body weight ratio is decreased in *Irs2*^-/- ^mice**. Kidney weights and kidney: body weight ratios in male and female mice at 13-14 wk. Click here for file
