# Supplementary material for: Deletion of Irs2 causes reduced kidney size in mice: role for inhibition of GSK3β?
Source: BMC Dev Biol. 2010 Jul 6;10:73. doi: 10.1186/1471-213X-10-73 (PMC2910663; doi:10.1186/1471-213X-10-73)
Supplement: Additional file 1 — Elevated IRS1 gene expression in the kidneys of male 13-14 wk Irs2-/- diabetic mice. Compensatory changes in kidney IRS1 in the absence of IRS2. [file 1471-213X-10-73-S1.PPT]

## Slide 1
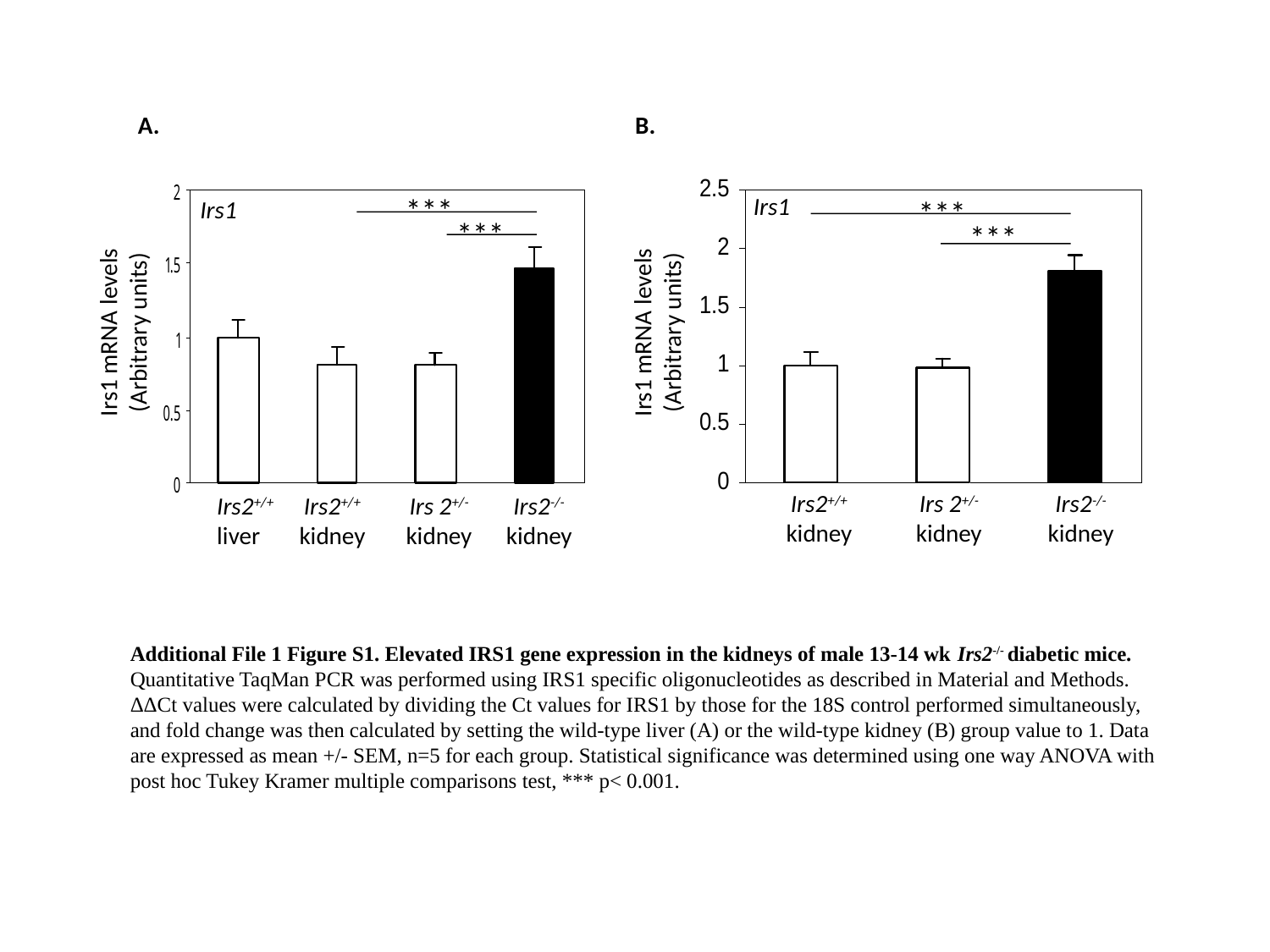

A.
B.
***
Irs1
***
Irs1
***
***
Irs1 mRNA levels
(Arbitrary units)
Irs1 mRNA levels
(Arbitrary units)
Irs2+/+
kidney
Irs 2+/-
kidney
Irs2-/-
kidney
Irs2+/+
liver
Irs2+/+
kidney
Irs 2+/-
kidney
Irs2-/-
kidney
Additional File 1 Figure S1. Elevated IRS1 gene expression in the kidneys of male 13-14 wk Irs2-/- diabetic mice.
Quantitative TaqMan PCR was performed using IRS1 specific oligonucleotides as described in Material and Methods.
ΔΔCt values were calculated by dividing the Ct values for IRS1 by those for the 18S control performed simultaneously,
and fold change was then calculated by setting the wild-type liver (A) or the wild-type kidney (B) group value to 1. Data
are expressed as mean +/- SEM, n=5 for each group. Statistical significance was determined using one way ANOVA with
post hoc Tukey Kramer multiple comparisons test, *** p< 0.001.
