# Supplementary material for: Deletion of Irs2 causes reduced kidney size in mice: role for inhibition of GSK3β?
Source: BMC Dev Biol. 2010 Jul 6;10:73. doi: 10.1186/1471-213X-10-73 (PMC2910663; doi:10.1186/1471-213X-10-73)
Supplement: Additional file 2 — Reduced kidney size is evident in Irs2-/- kidneys compared to wild-type at 12 days of age. Kidney size is reduced in Irs2-/- mice at 12 d of age. [file 1471-213X-10-73-S2.PPT]

## Slide 1
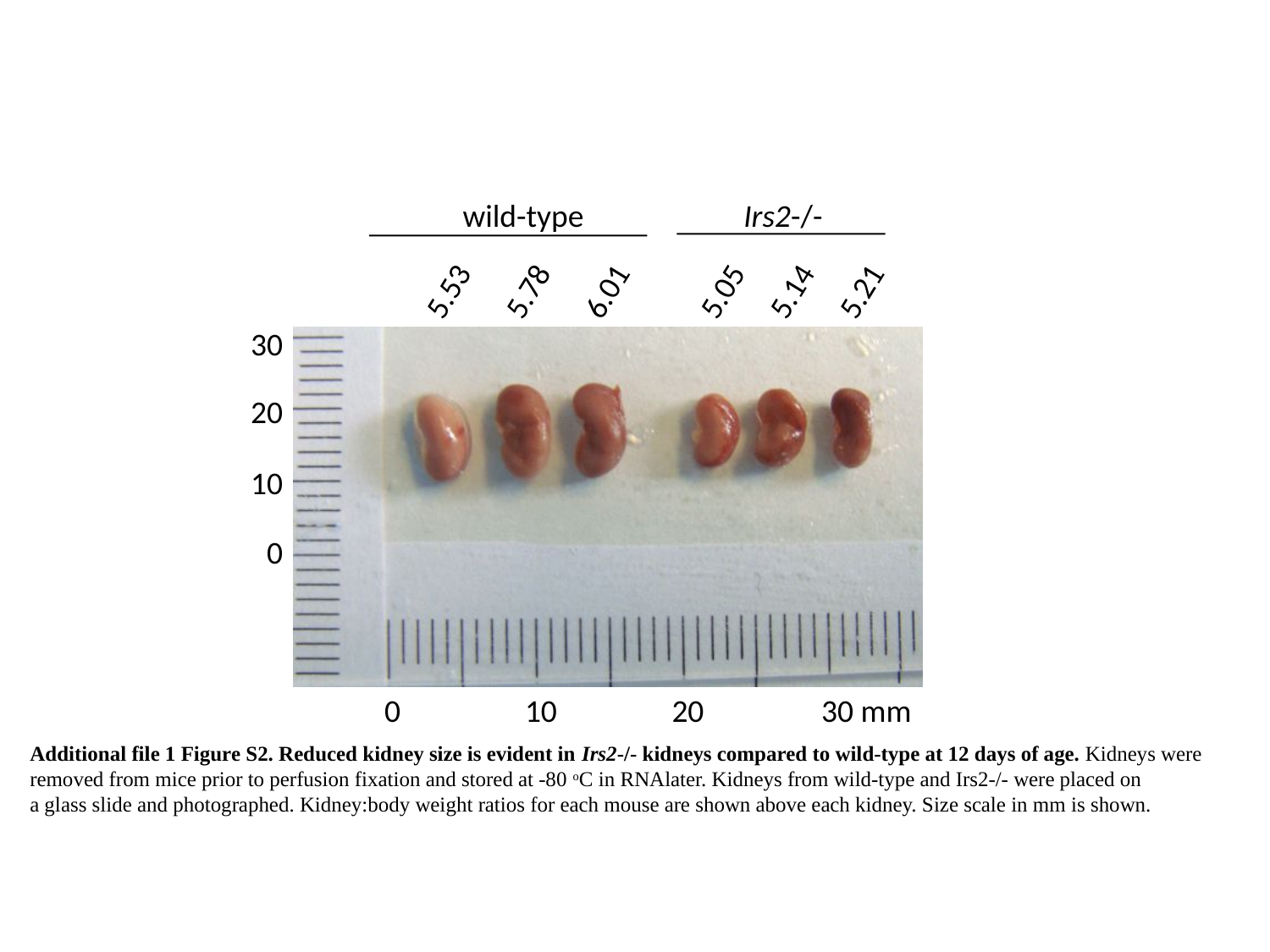

wild-type
Irs2-/-
5.53
5.78
6.01
5.05
5.14
5.21
30
20
10
0
0
10
20
30 mm
Additional file 1 Figure S2. Reduced kidney size is evident in Irs2-/- kidneys compared to wild-type at 12 days of age. Kidneys were
removed from mice prior to perfusion fixation and stored at -80 oC in RNAlater. Kidneys from wild-type and Irs2-/- were placed on
a glass slide and photographed. Kidney:body weight ratios for each mouse are shown above each kidney. Size scale in mm is shown.
