# Supplementary material for: Deletion of Irs2 causes reduced kidney size in mice: role for inhibition of GSK3β?
Source: BMC Dev Biol. 2010 Jul 6;10:73. doi: 10.1186/1471-213X-10-73 (PMC2910663; doi:10.1186/1471-213X-10-73)
Supplement: Additional file 3 — Irs2 deletion does not significantly alter gross kidney structure. Histology of Irs2-/- kidneys showing no significant changes in morphology. [file 1471-213X-10-73-S3.PDF]

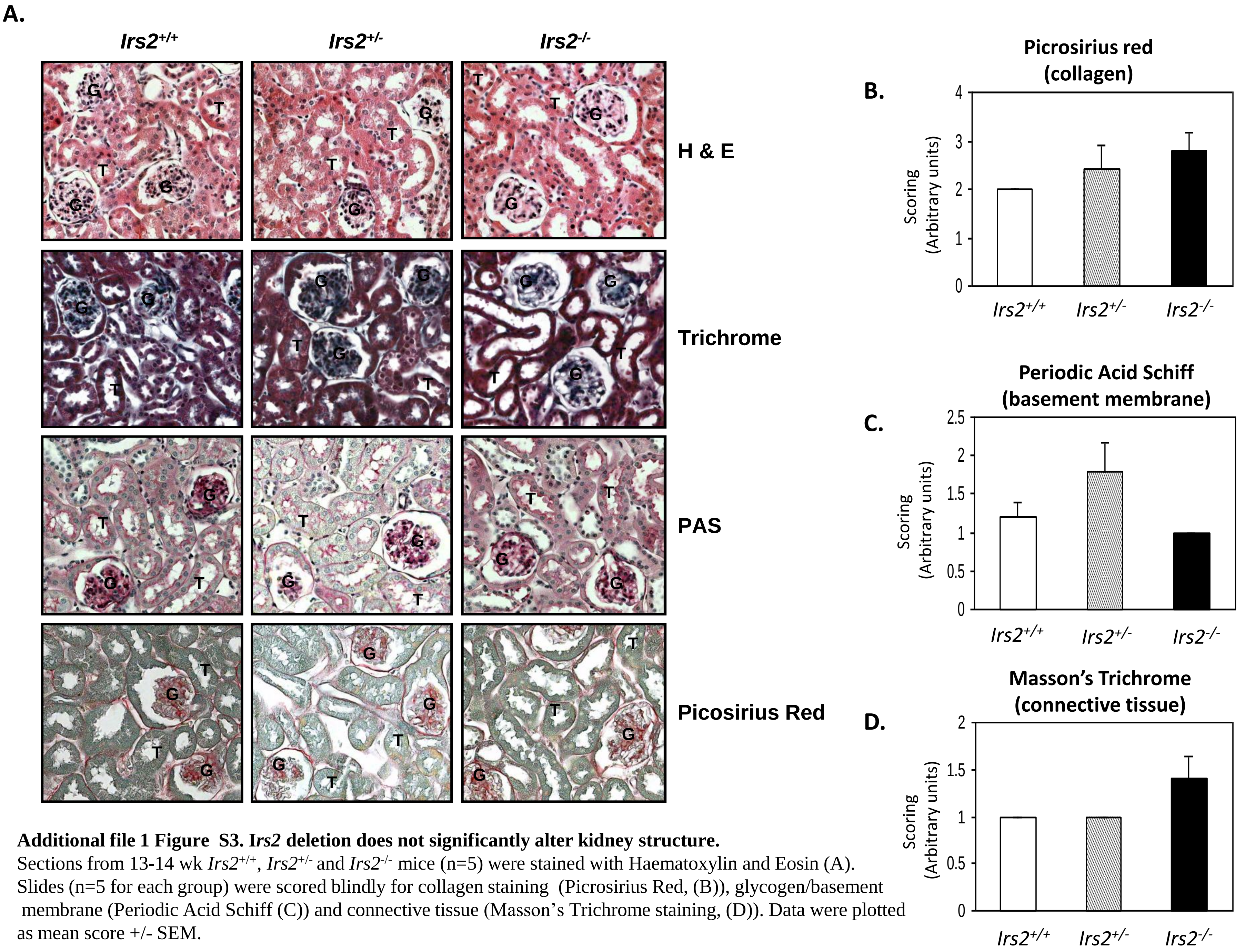

Additional file 1 Figure S3. I*rs2* deletion does not significantly alter kidney structure.
Sections from 13-14 wk *Irs2*$^{+/+}$, *Irs2*$^{+/-}$ and *Irs2*$^{-/-}$ mice (n=5) were stained with Haematoxylin and Eosin (A). Slides (n=5 for each group) were scored blindly for collagen staining (Picrosirius Red, (B)), glycogen/basement membrane (Periodic Acid Schiff (C)) and connective tissue (Masson's Trichrome staining, (D)). Data were plotted as mean score +/- SEM.
