# Supplementary material for: Deletion of Irs2 causes reduced kidney size in mice: role for inhibition of GSK3β?
Source: BMC Dev Biol. 2010 Jul 6;10:73. doi: 10.1186/1471-213X-10-73 (PMC2910663; doi:10.1186/1471-213X-10-73)
Supplement: Additional file 4 — Irs2-/- mice have reduced glomerular number but normal nephron density. No major changes in glomerular density in Irs2-/- kidneys [file 1471-213X-10-73-S4.PPT]

## Slide 1
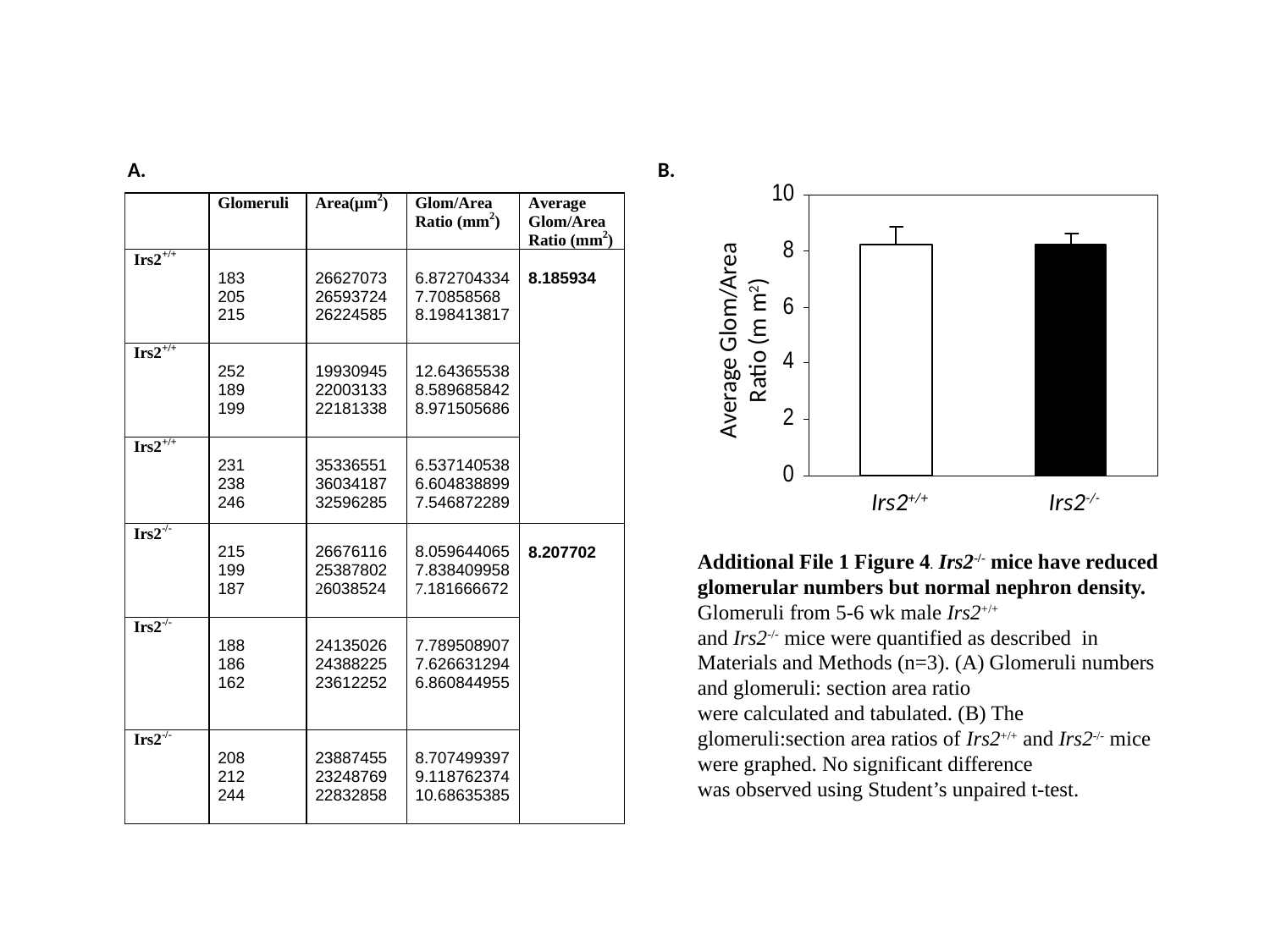

A.
B.
Average Glom/Area
Ratio (m m2)
Irs2+/+
Irs2-/-
Additional File 1 Figure 4. Irs2-/- mice have reduced glomerular numbers but normal nephron density. Glomeruli from 5-6 wk male Irs2+/+
and Irs2-/- mice were quantified as described in Materials and Methods (n=3). (A) Glomeruli numbers and glomeruli: section area ratio
were calculated and tabulated. (B) The glomeruli:section area ratios of Irs2+/+ and Irs2-/- mice were graphed. No significant difference
was observed using Student’s unpaired t-test.
