# Supplementary material for: Deletion of Irs2 causes reduced kidney size in mice: role for inhibition of GSK3β?
Source: BMC Dev Biol. 2010 Jul 6;10:73. doi: 10.1186/1471-213X-10-73 (PMC2910663; doi:10.1186/1471-213X-10-73)
Supplement: Additional file 5 — Kidney: body weight ratio is decreased in Irs2-/- mice. Kidney weights and kidney: body weight ratios in male and female mice at 13-14 wk. [file 1471-213X-10-73-S5.PPT]

## Slide 1
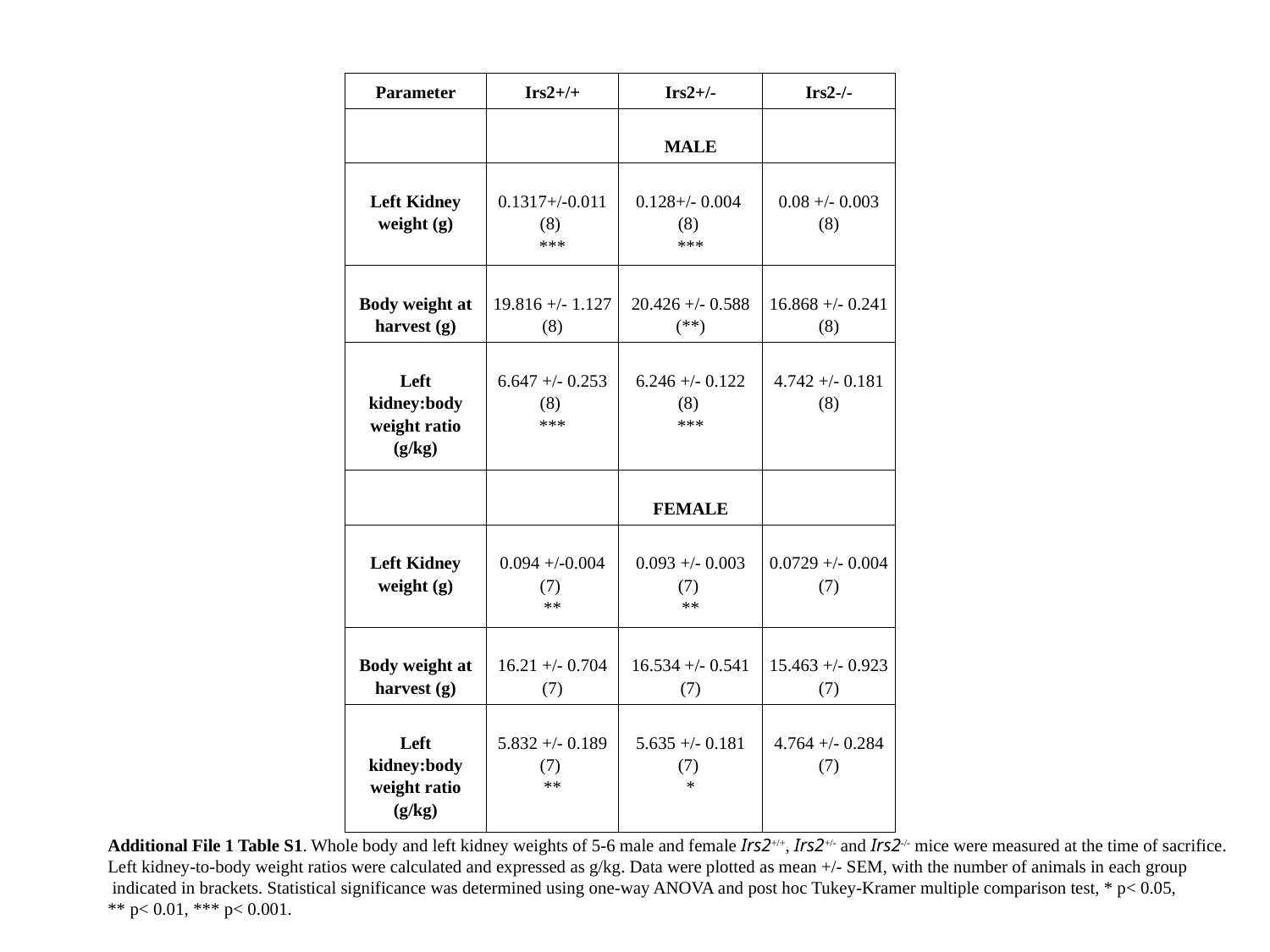

| Parameter | Irs2+/+ | Irs2+/- | Irs2-/- |
| --- | --- | --- | --- |
| | | MALE | |
| Left Kidney weight (g) | 0.1317+/-0.011 (8) \*\*\* | 0.128+/- 0.004 (8) \*\*\* | 0.08 +/- 0.003 (8) |
| Body weight at harvest (g) | 19.816 +/- 1.127 (8) | 20.426 +/- 0.588 (\*\*) | 16.868 +/- 0.241 (8) |
| Left kidney:body weight ratio (g/kg) | 6.647 +/- 0.253 (8) \*\*\* | 6.246 +/- 0.122 (8) \*\*\* | 4.742 +/- 0.181 (8) |
| | | FEMALE | |
| Left Kidney weight (g) | 0.094 +/-0.004 (7) \*\* | 0.093 +/- 0.003 (7) \*\* | 0.0729 +/- 0.004 (7) |
| Body weight at harvest (g) | 16.21 +/- 0.704 (7) | 16.534 +/- 0.541 (7) | 15.463 +/- 0.923 (7) |
| Left kidney:body weight ratio (g/kg) | 5.832 +/- 0.189 (7) \*\* | 5.635 +/- 0.181 (7) \* | 4.764 +/- 0.284 (7) |
Additional File 1 Table S1. Whole body and left kidney weights of 5-6 male and female Irs2+/+, Irs2+/- and Irs2-/- mice were measured at the time of sacrifice.
Left kidney-to-body weight ratios were calculated and expressed as g/kg. Data were plotted as mean +/- SEM, with the number of animals in each group
 indicated in brackets. Statistical significance was determined using one-way ANOVA and post hoc Tukey-Kramer multiple comparison test, * p< 0.05,
** p< 0.01, *** p< 0.001.
